# Expiratory time constants by electrical impedance tomography in hypoxemic and hypercapnic acute lung failure - a feasibility study

**DOI:** 10.1186/2197-425X-3-S1-A1000

**Published:** 2015-10-01

**Authors:** PL Róka, AD Waldmann, F Ender, SH Bohm, W Windisch, S Strassmann, C Karagiannidis

**Affiliations:** Budapest University of Technology and Economics, Budapest, Hungary; Swisstom AG, Landquart, Switzerland; Kliniken der Stadt Köln, Pneumology and Critical Care Medicine, Cologne, Germany; Witten/Herdecke University, Witten, Germany

## Introduction

During relaxed breathing expiration can be compared to a RC-circuit with *R* being the airway resistance, *C* the respiratory system compliance and *τ = R∙C* the expiratory time constant. For the adult respiratory system, the normal *τ* is around 0.8 s. Different lung pathologies have different time constants. Two common disease types are the acute respiratory distress syndrome (ARDS) and the chronic obstructive pulmonary disease (COPD). ARDS is characterized by stiff or noncompliant lungs with low compliance and normal or lower resistance which results in shorter *τ*. COPD is characterized by impaired airflow or high airways resistance and high compliance which results higher *τ*.

*τ* reveals information about respiratory mechanics and the time required for the lungs to empty. Traditional pulmonary function tests provide global information only. EIT is a non-invasive real-time imaging technology which determines changes of lung volumes on a regional basis assuming that local impedance changes are proportional to local changes in lung volume. Pikkemaat [1] introduced the method to calculate regional *τ*, what we improved and applied in 10 patients.

## Methods

Time constants were calculated from global and regional volume signals measured with EIT in 10 patients with hypoxemic (mainly ARDS) or hypercapnic (COPD) lung failure. Since the onset of expiration is mostly dominated by inertial effects it does not show an exponential behaviour [2], it is advised [3] to start analysis only at 75% of the signal amplitude. Skipping the first 25% of every signal's amplitude we analysed the global and regional temporal behaviour of each pixel until the end of their expiration of an EIT sequence by fitting in a nonlinear least square manner using MATLAB (The MathWorks, USA) an exponential curve whose decay provides the *τ*.

EIT data were measured with *Swisstom BB*^*2*^ (Swisstom AG, Switzerland) and the time constants of ensemble averaged of 10 consecutive breaths of 5 patients with COPD and 5 with ARDS were determined. Only *τ* values within the range of 0.05 and 5 sec stemming from curve fits with an R^2^ higher than 0.6 were considered thereby excluding poorly ventilated areas and those in which curve fitting was poor, see Table 1. Overall results for ARDS and COPD are expressed as mean ± SD.

## Results and Conclusions

In these 10 patients global *τ* was 0.56 ± 0.13 s in ARDS and 1.26 ± 0.29 s in COPD, and the mean of regional *τ* was 0.57 ± 0.12 s in ARDS and 1.17 ± 0.20 s in COPD. In ventilated areas their values and distribution were within the expected range. ARDS lungs were “faster” whereas COPD-lungs were “slower” reflecting the airway diseases. We showed that EIT can be used to determine global and regional time constants from passive exhalation and distinguish different lung pathologies.

**Figure 1:**
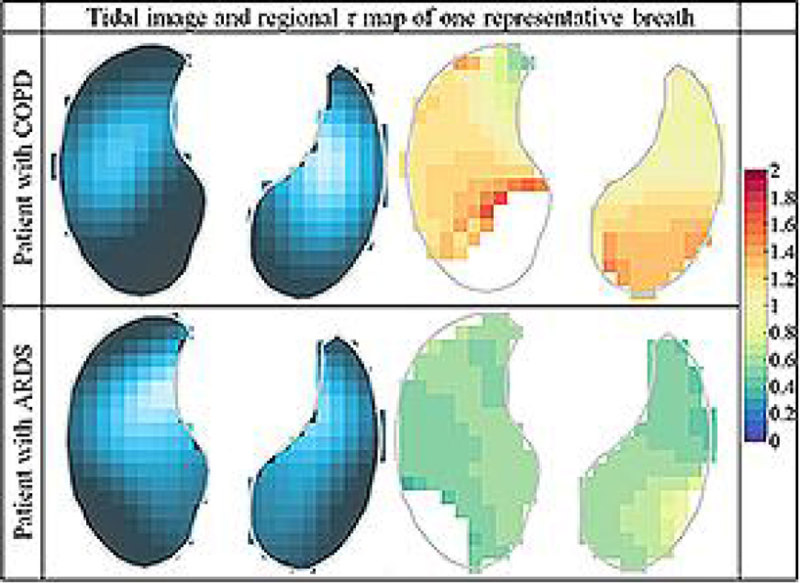
**[Table 1: Ventilation and τ distribution]**.
